# Exploring the Genome of Cheese Starter Lactic Acid Bacterium *Lactococcus lactis* subsp. *lactis* CECT 4433

**DOI:** 10.1128/genomeA.01142-14

**Published:** 2014-11-13

**Authors:** Diogo Antonio Tschoeke, Ana Paula B. Moreira, Luciane A. Chimetto Tonon, Milene Miranda A. de Mesquita, Gustavo B. Gregoracci, Bruno Gomez-Gil, Rogério Valle, Cristiane C. Thompson, Fabiano L. Thompson

**Affiliations:** a Instituto de Biologia, Universidade Federal do Rio de Janeiro (UFRJ), SAGE-COPPE, Rio de Janeiro, Brazil; b Fundação Oswaldo Cruz, Instituto Oswaldo Cruz, Laboratório de Vírus Respiratórios e do Sarampo, Rio de Janeiro, Brazil; c Universidade Federal de São Paulo, Campus Baixada Santista, Departamento de Ciências do Mar, Santos, Brazil; d A.C. Unidad Mazatlán, CIAD, Mazatlán, México; e SAGE-COPPE UFRJ, Rio de Janeiro, Brazil

## Abstract

Here, we present the draft genome sequences of *Lactococcus lactis* subsp. *lactis* CECT 4433, a cheese fermentation starter strain. The genome provides further insight into the genomic plasticity, biocomplexity (including gene strain specifics), and evolution of these genera.

## GENOME ANNOUNCEMENT

The *Lactococcus lactis* subsp. *lactis* strain CECT 4433 is a member of the lactic acid bacteria (LAB), known as cheese starters. Commonly, LAB is dominant in milk and fermented dairy products, comprising a heterogeneous group of microorganisms that convert carbohydrates into lactic acid (1, 2). LAB are widely distributed in nature and occur naturally as the indigenous microflora in fermented foods, and play a major role in flavors, texture, and preservative qualities (3).

Strains of *L. lactis* have fast growth and rapid production of lactic acid in milk (1). Many industrial and artisanal starter cultures use *L. lactis* to ferment dairy products, especially hard and semi-hard cheeses, and it plays a key role in determining shelf-life, preservation, and organoleptic quality and thus influences the quality and safety of these products. The growth of *L. lactis* in milk is associated with the rapid production of lactic acid, providing flavor, assisting in curd formation, preventing the growth of pathogenic and spoilage bacteria, and creating optimal biochemical conditions for ripening (2). Due to the availability for genetic engineering and genome sequencing efforts, *L. lactis* has become a model organism (4). For example, an ethanol pathway has also been introduced into LAB hosts in order to produce ethanol (5). Here, we present the genome sequence of the *L. lactis* subsp. *lactis* strain CECT 4433.

The genome sequences of *L. lactis* subsp. *lactis* strain CECT 4433 was sequenced with the Ion PGM system, as described previously by Gorriti et al. (6). The Ion PGM 200 sequencing kit was used for all sequencing reactions. The reads obtained were assembled using *de novo* assembly in the Ion Torrent Platform based on MIRA software (7). After the MIRA assembly, we performed a second assembly based on 224 MIRA contigs, using CAP3 software (8). The genome was annotated automatically using the RAST (Rapid Annotations Using Subsystems Technology) server (9). The size of the draft genome of *L. lactis* subsp. *lactis* CECT 4433 is 2,579,153 bp, comprising 111 contigs with a G+C content of 34.92% and 28× coverage. The *N*_50_ contig length was 44,334 bp, the largest contig length was 145,182 bp, and the smallest was 540 bp. The numbers of CDSs was 2,643 in *L. lactis* subsp. *lactis* CECT 4433; rRNA sequences found in *L. lactis* subsp. *lactis* CECT 4433 were 9: 6 copies of 5S rRNA, 2 copies of 16S rRNA, and one 23S rRNA gene. Furthermore, 58 tRNA sequences were found in CECT 4433. Comparing the sequence comparison of CECT 4433 and *L. lactis* subsp. *lactis* IL1403^T^, using the SEED comparative tool, we found 394 genes only in CECT 4433. We identified specific genes related to the carbohydrate metabolism as mannose-6-phosphate isomerase, glycosyltransferase, and sucrose-6-phosphate hydrolase and genes related to amino acid metabolism as NADP-specific glutamate dehydrogenase and arginine/ornithine antiporter ArcD. However, 47.96% (188/394) of these genes were annotated as hypothetical proteins or unknown or unnamed proteins and 20.3% (80/394) were annotated as phage associated proteins (e.g., phage tail fibers, capsid, and DNA invertase).

### Nucleotide sequence accession numbers.

This whole-genome shotgun project has been deposited at DDBJ/EMBL/GenBank under the accession no. JRKY00000000. The version described in this paper is version JRKY01000000.
